# Calycosin prevents NLRP3-induced gut fibrosis by regulating IL-33/ST2 axis

**DOI:** 10.1016/j.heliyon.2024.e30240

**Published:** 2024-04-25

**Authors:** Xiujun Liao, Haiting Xie, Saojun Yu

**Affiliations:** Department of Colorectal Surgery and Oncology, Key Laboratory of Cancer Prevention and Intervention, Ministry of Education, The Second Affiliated Hospital, Zhejiang University School of Medicine, Hangzhou, Zhejiang, 310058, China

**Keywords:** Inflammatory bowel disease, Intestinal interstitial fibrosis, Calycosin, NLRP3, IL-33

## Abstract

Intestinal interstitial fibrosis is a core event of inflammatory bowel disease (IBD) development. Calycosin has been recognized to carry various therapeutic bioactivities. However, the role of calycosin in intestinal interstitial fibrosis remains to be illustrated. This aim of this study was to explore the effects of calycosin on intestinal interstitial fibrosis in IBD and the underlying mechanisms. The *in vitro* and in vivo models were established by using TNBS-induced mouse IBD model and co-culture of intestinal epithelial cells and intestinal interstitial cells; moreover, lentivirus-mediated knockdown of NLRP3 expression was applied. The results showed that calycosin significantly improved the intestinal interstitial fibrosis of TNBS-induced IBD. Mechanistically, calycosin downregulated NLRP3 expression and inhibited the activation of IL-33/ST2 signaling in intestinal epithelial cells, which subsequently impedes intestinal interstitial cell migration and activation by regulating the secretion of IL-33/ST2 signaling-induced fibrosis mediators. Notably, combination of calycosin and NLRP3 signaling blockade improved the intestinal interstitial fibrosis extent. Altogether, this study suggests calycosin can improve intestinal interstitial fibrosis by downregulating NLRP3-IL-33/ST2 signaling, reducing inflammation and decreasing pro-fibrotic factors’ secretion, which provides a new perspective for therapeutic options of IBD.

## 1 Introduction

Inflammatory bowel disease (IBD) is a kind of chronic non-specific intestinal inflammatory disease, with a significant upward trend in these years [1]. In the pathogenesis of IBD, long-term chronic inflammatory stimulation leads to excessive proliferation of intestinal interstitial cells, promotes the extracellular matrix (ECM) deposition in the intestinal wall, and accelerates the growth of the intestinal muscle layer, which ultimately leads to intestinal fibrosis, intestinal stenosis, and loss of intestinal wall compliance; thus, aggravating symptoms including diarrhea and abdominal pain, forming fistula, and even malignant transformation [1,2]. Although the current biological agents targeting the pathogenesis of IBD have achieved definite efficacy in controlling disease activity and maintaining disease remission [3], the long-term outcome of IBD patients is not satisfying. In contrast, traditional medicine and natural compounds have shown advantages of precise efficacy and high safety in the clinical treatment of IBD [[4], [5], [6], [7]]. It is well-known that the traditional herb Radix Astragali (RA) has been used for decades as an immunomodulatory drug to treat various inflammatory diseases [8,9]. Calycosin is the most potent component of RA and exhibits multiple biological properties including antioxidant, antiinflammation, neuroprotection, and etc. [[10], [11], [12]].

Inflammation-driven intestinal fibrosis has been recognized as the key characteristic of IBD [13]; clarifying the inflammatory characteristics of intestinal fibrosis in IBD may become a breakthrough to improve the current treatment dilemma. Recently, it has been suggested that calycosin can inhibit intestinal fibrosis to a certain extent by improving blood circulation and regulating the inflammation process [14], implying the possibility of calycosin treating IBD. Inflammasome has been shown to modulate the inflammatory response and is attracting more and more attention in intestinal fibrosis [15]. Previous studies have shown that NLRP3 inflammasome is activated in a variety of intestinal lesions, which participates in the development of intestinal interstitial fibrosis through mediating inflammation; relatively speaking, the inhibition of NLRP3 inflammasome activation can reduce ROS production and relieve intestinal fibrosis [16,17]. All of these studies imply the potential association between calycosin treatment and the activation of NLRP3 inflammasome, which may be a criticl pathogenic link in understanding the development of intestinal fibrosis in IBD. However, the underlying mechanism are still unknown. Hence, this study aimed to investigate the effect of calycosin on the pathogenesis of IBD and its molecular mechanism, based on both *in vitro* and in vivo models. The study will help to understand the pathogenesis of IBD and develop some new therapeutic drugs.

## 2 Methods

### 2.1 Grouping of experimental animals and establishment of IBD animal model

BALB/C mice (body weight 18–21g, 8–10 weeks, clean grade) were purchased from Zhejiang Vital River Laboratory Animal Technology Co., Ltd (Hangzhou, China) and were adaptively fed for 1 week; IBD was induced by 2,4,6-trinitrobenzenesulfonic acid (TNBS; CAS#2508-19-2*, Invitrogen, USA) in these mice as described elsewhere [18]. In brief, these mice were given 1.5–2.5 mg of TNBS with 45 % ethanol solution, a total of 100 μl enema solution. The control group received normal saline enema once a week for 6 weeks. After successful modeling, regular food was given to these mice. The calycosin, purchased from Mansite Biotechnology with an HPLC purity of ≥99.89 % (Chengdu, China), was administered by gavage every day at a dose of 10 mg/kg for a period of 4 weeks. At the same time, a control group was established which received normal saline. In addition, to observe the efficacy of NLRP3 inhibitor-MCC950 and calycosin combination therapy in treating intestinal interstitial fibrosis, we treated the IBD model mice with MCC950 (i.p., 10 mg/kg, 3 times/week) or calycosin single use, the combination of MCC950 and calycosin; afterthought, the extent of intestinal interstitial fibrosis and pathohistological manifestations as examined by performing a HE staining, Masson staining and IHC staining; meanwhile the serum levels of liver function markers including ALT and AST were examined according to the previously published protocol [19].

### 2.2 Intestinal histopathological examination of model animals

The mental status, hair softness, stool characteristics and weight changes of mice were observed and recorded every day. According to the commonly used scoring standard of colon histomorphology, the general damage of the colon in mice was scored; after routine dewaxing, staining and other pathological treatments, the histopathological changes of colon tissue were observed by using a light microscope. The histopathology of the mouse colon was evaluated and scored according to the scoring standard [20], and the expression of fibrosis-related molecules was further detected by performing an immunohistochemistry assay.

### 2.3 Isolation and culture of primary murine intestinal epithelial cells

Isolation and culture of primary murine intestinal epithelial cells was referenced to the previously published protocol [21]. The small intestinal tissue was collected and cut into 3–4 cm pieces in order to isolate primary intestinal epithelial cells. The inverted tissue was mounted on an inoculation loop, incubated for 10 min at 37 °C in 30 mM EDTA (Thermo Fisher Scientific, USA) and homogenized by utilizing a biovortexer (Sigma, USA) with 10–12 pulses/1–2 s duration. Epithelial cell debris was separated by triple sedimentation at 1×*g* at 4 °C for 20 min, resulting finally in producing E-calcine-positive epithelial cells with a purity of 85–90 %. The epithelial cells were suspended in DMEM complete growth media, plated in a 12-well cell culture plate at a density of 1 × 10^6^ cells/mL and 50 % media changes were conducted every 3 days thereafter.

### 2.4 Cell culture

To construct the co-culture system *in vitro*, immortalized mouse intestinal epithelial cells MODE-K and mouse small intestine mesenchymal cells Cajal were purchased from Procell Life Science&Technology Co.,Ltd. (Wuhan, Hubei, China) and were cultured in DMEM (Hyclone, Hangzhou, China) supplemented with 10 % FBS, 2 mM l-glutamine, 1 % sodium pyruvate and 100 units/mL penicillin G, 100 μg/mL streptomycin sulfate (Beyotime, Hangzhou, China). Cajal cells were treated with calycosin treatment (40 μM for indicated time). Moreover, MODE-K cells with IL-33 knockdown followed by NLRP3 overexpression were co-cultured with Cajal cells. All cells were cultured in a humidified atmosphere of 5 % CO2 at 37 °C.

### 2.5 Immunofluorescence staining

The intestinal tissue samples were cut into the pieces of 5mm × 5 mm and fixed overnight in 4 % Paraformaldehyde (PFA) fix solution. The fixed intestinal tissues were embedded in paraffin, and cut into sequential 8-μm thick slides. The standard protocol of the immunofluorescent staining was performed according to the report [22]. Collagen-I (1:100, ab270993, Abcam, USA) staining area and extent were assessed under microscope examination. The quantitative analyses was performed by using the software (LAS X Core Software, Leica Microsystems, GER).

### 2.6 Western blotting

Western blotting was performed as previously described [23]. Molecular expression of NLRP3, ASC, TIMP-1, α-SMA and collagen I proteins in intestinal tissues and cells were analyzed using western blotting. The proteins were extracted from intestinal tissues or MODE-K or Cajal cells by RIPA lysis buffer (Beyotime, China) and the concentration was determined by using a bicinchoninic acid protein assay kit (Beyotime, China). Equal amounts (20 μg) of proteins for each sample were loaded onto 10 % SDS-PAGE to separate proteins. Proteins were wet transferred to a PVDF membrane (Thermo Fisher Scientific, USA), which was blocked by incubation with 5 % skim milk (Beyotime, Hangzhou, China) for 2 h at room temperature. Whereafter, the membrane was incubated with primary antibodies (NLRP3, 1:1000, ab272702, Abcam, USA; ASC, 1:1000, ab47092, Abcam, USA; TIMP-1, 1:1000, ab179580, Abcam, USA; α-SMA, 1:1000, ab232784, Abcam, USA and collagen I, 1:1000, ab270993, Abcam, USA) at 4 °C overnight. After pouring off first antibodies, the membrane was rinsed briefly with TBST buffer (Beyotime, Hangzhou, China) three times and incubated with an appropriate secondary antibody for 2h. Blots were visualized using the SuperSignal Chemiluminescent Substrate (Thermo Fisher Scientific Inc. USA). Results were shown after the normalization of the loading amounts in each lane by GAPDH (1:5000, ab181602, Abcam, USA). All experiments were performed 3 times. The Western blot bands were quantitatively analyzed by using the software Image J.

### 2.7 Real-time PCR

Real-time PCR was carried out as previously described [24]. Total RNA was extracted from intestinal tissues or the cultured cells using TRIzol reagent (Life Technologies, USA). The cDNAs of genes including *nlrp3*, *α-SMA*, *collagen-1* and *Kgf*, *ctg*, *fgf2*, *tgf-β*, *Igf-1* and *pdgf-b* were obtained by using the Transcriptor First Strand cDNA Synthesis Kit combined with FastStart Universal SYBR Green Master (Roche, Basel, Switzerland). All of the reactions were performed in triplicate in a real-time PCR system (ABI 7500, Applied Biosystems, USA), and GAPDH was set as an internal control. The 2^−ΔΔ^Ct method was applied for the data analysis. The details of primers used in the study were listed in Table 1. Moreover, a mouse Inflammatory Cytokines & Receptors PCR Array containing 84 different inflammation-related cytokines and receptors was used to elucidate how calycosin can improve intestinal interstitial fibrosis in IBD animal models. The detection was performed by using a WCGENE® PCR Array Plate. 84 genes assayed on the PCR Array were listed in Supplemental Data Table S1. The transcriptional level of each gene were normalized based on those in the control. A list of genes with ≥2.0 fold changes were identified, based on a Welch *t*-test (P < 0.05).

**Table 1:** Primers sequences used for real time PCR.

Gene	Accession number	Forward (5′-3′)	Reverse (5′-3′)	Primer efficiency(%)	Annealing temperature	Product size (bp)
GAPDH	NM_001411840.1	CAACTACATGGTCTACATGTTC	CTCGCTCCTGGAAGATG	96.8	55 °C	132
NLRP3	NM_001359638.1	ATTACCCGCCCGAGAAAGG	TCGCAGCAAAGATCCACACAG	96.3	60 °C	188
IL-1b	NM_008361.4	GCAACTGTTCCTGAACTCAACT	ATCTTTTGGGGTCCGTCAACT	100.3	58 °C	136
caspase-1	NM_009807.2	CCAGAGCACAAGACTTCTGAC	TGGTGTTGAAGAGCAGAAAGC	98.5	55 °C	129
a-SMA	NM_007392.3	GCAATGAAGACGAAGGCGAC	GTGCCCGTGCGAGATTTTAG	99.3	56 °C	148
TIMP-1	NM_001044384.2	TGCCACCTTTTGACAGTGATG	TGATACTGCCTGCCTGAAGC	100.4	55 °C	172
Collagen I	NM_007742.4	TGATACTGCCTGCCTGAAGC	CAC ACT CGG ACC ACA TCC TT	99.3	58 °C	135

### 2.8 Immunohistochemistry (IHC)

Immunohistochemistry was performed as described previously [25]. Briefly, formalin-fixed paraffin-embedded intestinal tissues from model mice were sectioned into 4-μm slices. After dewaxing, hydration and antigen heat retrieval, slices were blocked with 10 % donkey serum and incubated overnight at 4 °C with primary antibodies as follows: anti-α-SMA (1:100; ab232784, Abcam, USA) and anti-collagen I (1:100; ab270993, Abcam, USA) diluted in TBS containing 2 % bovine serum albumin (BSA, Beyotime, Hangzhou, China). After subsequently wash, these slices were incubated with HRP-conjugated goat anti-rabbit IgG secondary antibody (1:100; ab6728, Abcam, USA). Following by three times washing, slices were incubated for 10 min with 3, 3′-diaminobenzidine tetrachloride (Beyotime, Hangzhou, China) and specific staining was visualized by a light microscopy. Images were acquired under × 100 field with Leica MDi8 inverted microscope (Leica Microsystems, GER). Quantitative expression of immunostaining was determined at a fixed threshold using ImageJ software (Maryland, USA).

### 2.9 ELISA (enzyme-linked immunosorbent assay)

The filtered supernatant was used to determine the levels of various cytokines by associated ELISA kits according to the manufacturer's instructions. Mouse IL-33 ELISA kit (*Cat no*.ELM-IL33-1), IL-33 ELISA kit (*Cat no.* ELM-IL13-1), IL-4 ELISA kit (*Cat no.* IQM-IL4-1), IL-10 ELISA kit (*Cat no.* IQM-IL10-1), TNF-β ELISA kit (*Cat no.* ELM-TNFB-1) were purchased from RayBiotech, USA and mouse TGF-β ELISA kit (*Cat no.* MBS824944) was from MyBioSource, USA. In brief, 96-well ELISA plates coated with anti-mouse monoclonal antibodies were blocked with 200 μL per well of assay diluent for 2h at room temperature; then, 100 μL media samples was added into each well and incubated for the indicated time. Afterward, a 100 μL diluent of secondary antibody was added and incubated for 30 min at room temperature; TMB substrates solution was added and incubated in the dark for 30 min, and the reaction was stopped with stopping solution. The values of densities were read at 450 nm.

### 2.10 Construction of vectors and lentivirus transfection

Cell transfection was performed according to our previous study [26]. In detail, the lentiviral vectors containing NLRP3 (LV-NLRP3 or LV-shNLRP3) or IL-33 (LV-shIL33) or control were procured from HANBIO Biotech (Shanghai, China). The complete nucleotide sequence of each retroviral vector was confirmed before use. Cells were cultured to a confluence of 60 % and then exchanged into Opti-MEM (Thermo Fisher Scientific, USA) with lentiviral vectors (MOI ≈30) and 5 μg/ml polybrene. After incubation for 48 h, the transfection efficiency was evaluated under an inverted microscope; meanwhile puromycin (1 μg/ml) was used to eliminate the untransfected cells. Whereafter, the transfected cells were maintained in a fresh medium and harvested at the indicated times for subsequent real-time PCR and western blotting analysis.

### 2.11 Statistical analysis

Statistical analysis was performed with SPSS 19.0 software (SPSS, Chicago, IL, USA). were Mean ± SEM was used for continuous variables, whose comparison was carried out by using Wilcoxon test. Frequency (n) and proportion (%) were utilized for categorized variables, whose comparison was performed by using ANOVA. The comparisons among various groups were based on an independent *t*-test. All tests were two-sided and statistical significance was considered as P < 0.05. A majority of experiments were run in triplicate.

## 3 Results

### 3.1 Calycosin improves TNBS-Induced IBD

**Fig. 1:**
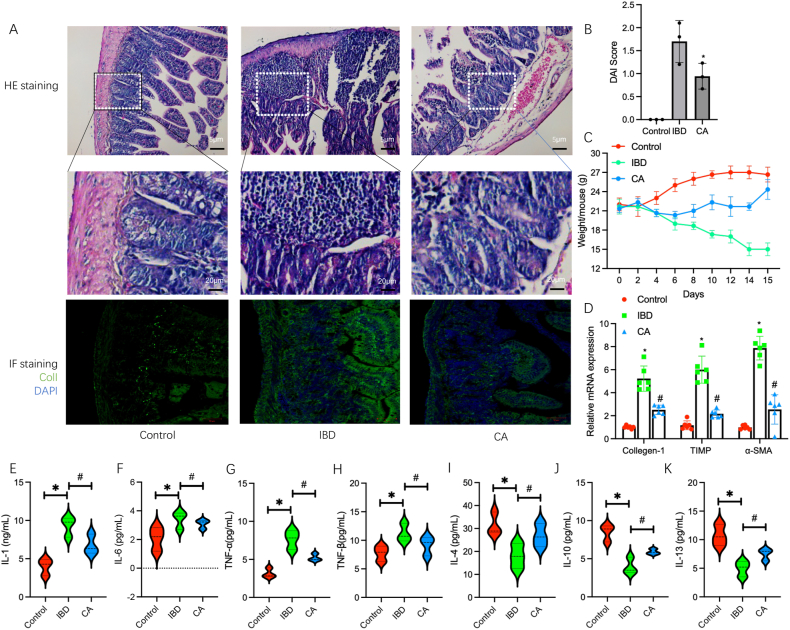
**Calycosin improves TNBS-Induced IBD**. (A) Representative images of HE staining and Collagen-I immunofluorescence staining in the intestinal tissues of control, IBD and CA-treated model animals at a magnification of × 200 and then representative fields were chosen for presentation. (B) The DAI (disease activity index) score of control, IBD and CA-treated model animals was analyzed (n = 3). (C) The weight of control, IBD and CA-treated model animals was counted. (D) The relative mRNA levels of fibrogenic factors including Collagen-1, TIMP and α-SMA in intestinal tissues from various groups were detected by using a qRT-PCR assay (n = 6). (E–K) The relative levels of pro-inflammatory cytokines including IL-1, IL-8, IL-33, TNF-α, TGF-β1 and anti-inflammatory cytokines including IL-4, IL-10 and IL-13 were decreased by using a ELISA assay (n = 6). Error bars indicate SEM. * indicates P < 0.05 vs. control groups.

To explore the potential protective effects of calycosin on IBD, we established a TNBS-induced IBD murine model and performed a histopathologic evaluation by using HE staining combined with Collagen I Immunofluorescence staining. The result showed that a heavy infiltration of inflammatory cells including macrophages, lymphocytes, and neutrophils, alongwith the formation of intestinal fibers and collagen deposition was observed in IBD model group. It was also observed that calycosin treatment significantly led to a considerable reduction in the effects of TNBS on the formation of intestinal fibers, collagen deposition, and infiltration of inflammatory cells in these model mice (Fig. 1A). Of note, the DAI (disease activity index) score and mouse weight in the CA-treated groups were significantly improved, compared to the model group (Fig. 1B-C). Moreover, the mRNA levels of some fibrogenic factors including Collagen-1, TIMP and α-SMA [27] in intestinal tissues from control group, IBD model group and CA-treated group were detected. The result showed that these factors were significantly increased in animal models, compared to controls (all P < 0.05); however, in the CA-treated group, these fibrogenic factors were significantly decreased, compared to the IBD model group (all P < 0.05) (Fig. 1D). In addition, the inflammation features in intestinal tissues from these groups above were evaluated by using ELISA assay. The result showed that, in IBD model group, the levels of pro-inflammatory cytokines including IL-1, IL-8, IL-33 and TNF-α, TGF-β1 [28] were significantly increased while anti-inflammatory cytokines including IL-4, IL-10 and IL-13 [28] were decreased (all P < 0.05). Furthermore, it also showed that, after calycosin treatment, the increase of pro-inflammatory cytokines was obviously inhibited and the decrease of anti-inflammatory cytokines was significantly improved in these model mice (all P < 0.05) (Fig. 1E-K). These results demonstrated that calycosin can attenuate intestinal interstitial fibrosis and improve the inflammation status in TNBS-induced IBD model mice.

### 3.2 Calycosin inhibits NLRP3-induced inflammation in IBD

**Fig. 2:**
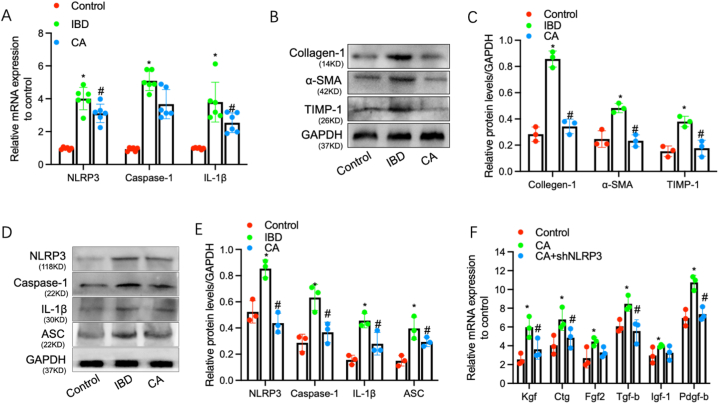
**Calycosin inhibits NLRP3-induced inflammation in IBD**. (A) The relative mRNA levels of NLRP3 and its downstream molecules including caspase-1, IL-1β were detected by using a qRT-PCR assay (n = 6). (B–C) The relative protein levels of Collagen I, TIMP-1 and α-SMA were detected in the isolated primary murine intestinal epithelial cells from the three groups by carrying out a Western Blotting assay (n = 3). (D–E) The relative protein levels of NLRP3, caspase-1 and IL-1β were detected in the isolated primary murine intestinal epithelial cells from the three groups by carrying out a western blotting assay (n = 3). (F) The relative mRNA levels of some fibrogenic factors including *Kgf, Ctg, Fgf2, Tgf-beta, Igf-1* and *Pdgf-b* in MODE-K cells with shNLRP3 or CA treatment were detected by qRT-PCR assay (n = 6). Error bars indicate SEM. * indicates P < 0.05 vs. control groups. # indicates P < 0.05 vs. CA groups.

As mentioned above, the finding suggests that inflammation is the core in the development of intestinal interstitial fibrosis in IBD [29]. To reveal the inflammatory mechanism by which calycosin is involved in intestinal interstitial fibrosis, we firstly evaluated the NLRP3 expression in a variety of animal groups including control group, IBD model group and CA-treated group. The result showed that the mRNA levels of NLRP3 and its downstream caspase-1 and IL-1β significantly increased in IBD model group, compared to controls; while in CA-treated group, the mRNA levels of the three factors were obviously downregulated, compared to IBD model group (Fig. 2A). Additionally, expressions of Collagen-1, TIMP-1 and α-SMA were detected in the isolated primary murine intestinal epithelial cells from the three groups. The result showed that such three proteins were significantly increased in intestinal epithelial cells from the IBD model animals and downregulated in intestinal epithelial cells from the CA-treated group, compared to the IBD model group (Fig. 2B-C). The result also showed that the expression levels of NLRP3 inflammasome-related factors including NLRP3, caspase-1 and ASC displayed similar significant changes (Fig. 2D-E). Moreover, to elucidate the role of NLRP3 in the stimulation of inflammation by calycosin, murine intestinal epithelial cell line MODE-K was used and lentivirus-mediated NLRP3 knockdown was carried out. The result showed that calycosin induced the expressions of some fibrogenic factors including *Kgf, Ctg, Fgf2, Tgf-beta, Igf-1* and *Pdgf-b* in MODE-K cells; however, this upregulation effect was inhibited after NLRP3 knockdown in MODE-K cells (Fig. 2F). These results suggested that the effect of calycosin on expression of these fibrogenic factors was NLRP3-dependent.

### 3.3 Calycosin regulates NLRP3 activation and its downstream IL-33/ST2 signaling in intestinal epithelial cells

**Fig. 3:**
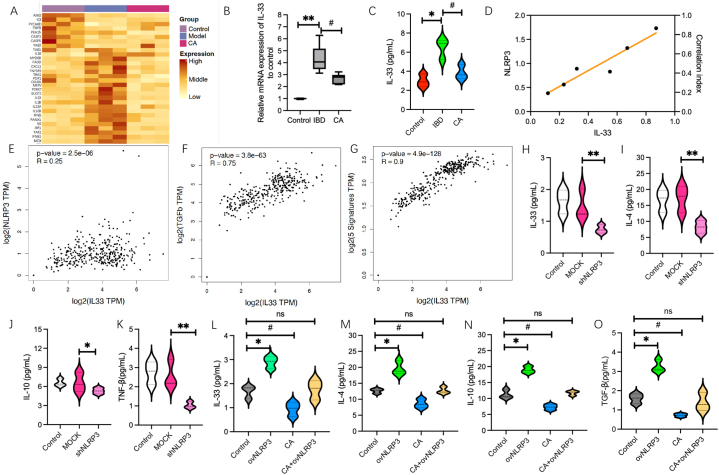
**Calycosin regulates NLRP3 activation and its downstream IL-33/ST2 signaling in intestinal epithelial cells**. (A) A mouse Inflammatory Cytokines & Receptors PCR Array containing 84 inflammation-related cytokines and receptors (YingBiotech Corp., shanghai) was used to compare and analyze the changes in the inflammation profile of the intestinal tissues from control, IBD and CA-treated model animals. (B–C) The relative mRNA and protein levels of IL-33 was detected by using qRT-PCR assay or Western Blotting assay, respectively. (D) The correlation between IL-33 and NLRP3 expression was analyzed in the intestinal tissues (n = 6). (E–G) The correlation between IL-33 expression and NLRP3 expression, pro-fibrotic factor TGF-β, pro-inflammatory factors signature in gastric tissue was analyzed by utilizing the GEPIA2 database. (H–K) The relative protein levels of IL-33 and IL-4, IL-13, TGF-β in MODE-K cells with NLRP3 knockdown were determined by using ELISA assay. (L–O) The relative protein levels of IL-33 and IL-4, IL-13, TGF-β in MODE-K cells with NLRP3 overexpression followed by CA treatment were determined by using an ELISA assay. Error bars indicate SEM. * indicates P < 0.05 vs. control groups. ** indicates P < 0.01 vs. MOCK groups. ^#^ indicates P < 0.01 in CA vs control.

To elucidate how calycosin can improve intestinal interstitial fibrosis in animal models of IBD, we used a mouse Inflammatory Cytokines & Receptors PCR Array containing 84 different inflammation-related cytokines and receptors to analyze the differences among the groups. The results showed that IL-33 was significantly and dominantly downregulated in the calycosin-treated group when compared to the IBD model group (P < 0.05) (Fig. 3A-C). Moreover, both in the model group and calycosin-treated group, IL-33 displayed a significantly positive correlation with NLRP3 expression (P < 0.05) (Fig. 3D). The correlation between IL-33 and NLRP3 expression was further analyzed by using the GEPIA2 database. The result showed that IL-33 expression was positively correlated with NLRP3 expression and pro-fibrotic factor TGF-β, pro-inflammatory factors in intestinal tissues (Fig. 3E-G). In addition, to verify the effect of NLRP3 on IL-33, NLRP3 was knocked down *in vitro* in MODE-K cells. The result showed that IL-33 expression was significantly inhibited and some pro-inflammatory and pro-fibrotic factors, such as IL-4, TGF-β and IL-13 were also obviously reduced (P < 0.05) (Fig. 3H-K). Notably, when MODE-K cells with NLRP3 overexpression were treated with calycosin, there was no significant effect on the expression of IL-33 or the levels of IL-4, IL-13, and TGF-β. However, when parental MODE-K cells were treated with calycosin, there was a significant decrease in both the expression and secretion of IL-33, as well as the levels of IL-4, IL-13, and TGF-β (P < 0.05) (Fig. 3L–O). The results indicated that calycosin-induced the downregulation of NLRP3 can impede the activation of IL-33/ST2 signaling and finally reduce inflammation and the secretion of pro-fibrotic factors.

### 3.4 NLRP3-IL-33/ST2 signaling in intestinal epithelial cells promotes activation and fibrosis-related factors of intestinal interstitial cells

**Fig. 4:**
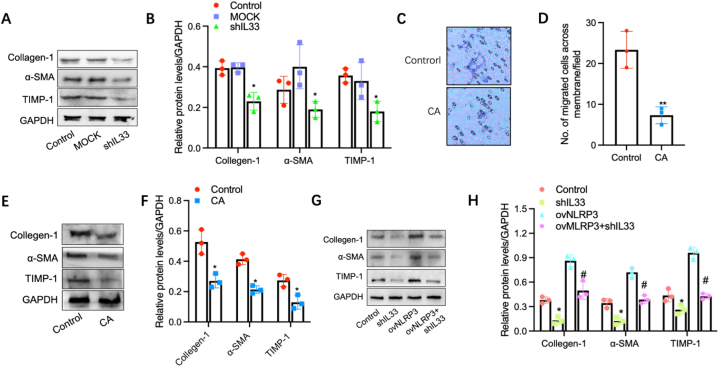
**NLRP3-IL-33/ST2 signaling in intestinal epithelial cells promotes activation and fibrosis-related factors of intestinal stromal cells**. (A–B) The relative protein levels of Collagen-1, TIMP and α-SMA in Cajal cells cultured with the media from MODE-K cells with IL-33 knockdown (Control: MODE-K cells; MOCK: MODE-K cells transfected with negative control shRNA; shIL33: MODE-K cells transfected with specific shRNA against IL33) were detected by carrying out a Western Blotting assay (n = 3). (C–D) The no. of Cajal cells across the membrane was counted by performing a Transwell assay (n = 3). (E–F) The relative levels of α-SMA and collagen-I expression in CA-treated Cajal cells were detected by carrying out a Western Blotting assay (n = 3). (G–H) The relative expressions of Collagen-1, TIMP and α-SMA in Cajal cells with shIL33, ovNLRP3 or combination were detected by carrying out a Western Blotting assay (n = 3). Error bars indicate SEM. * indicates P < 0.05 vs. controls.

It is well-known that intestinal stromal cell activation is the core event in intestinal interstitial fibrosis [30]; meanwhile based on the finding above in our study, we speculated there may be a potential connect between intestinal stromal cells and NLRP3-IL-33/ST2 signaling. To explore the relationship between NLRP3-IL-33/ST2 signaling activation and intestinal interstitial fibrosis, we detected some fibrosis-related factors including Collagen-1, TIMP and α-SMA in Cajal cells cultured with the media from MODE-K cells with IL-33 knockdown. The result showed that after IL-33 knockdown in MODE-K cells, the levels of these factors mentioned above in Cajal cells were significantly decreased (Fig. 4A-B). Moreover, compared to controls, calycosin treatment reduced the number of Cajal cells across the membrane, as indicated by the transwell assay (Fig. 4C-D). In addition, calycosin inhibited α-SMA and collagen-I expression in Cajal cells, suggesting the inhibition of Cajal cells activation (Fig. 4E-F). Furthermore, MODE-K cells with IL-33 knockdown followed by NLRP3 overexpression were co-cultured with Cajal cells. It was observed that NLRP3 overexpression upregulated the expressions of Collagen-1, TIMP and α-SMA in Cajal cells, but after IL-33 knockdown, the upregulation of Cajal cells activation was significantly inhibited (Fig. 4G-H). Altogether, these results indicated that NLRP3-IL-33/ST2 signaling plays an important role in calycosin promoting migration and activation of interstitial cells.

### 3.5 Combination of calycosin and NLRP3 inhibitor treating IBD in vivo

**Fig. 5:**
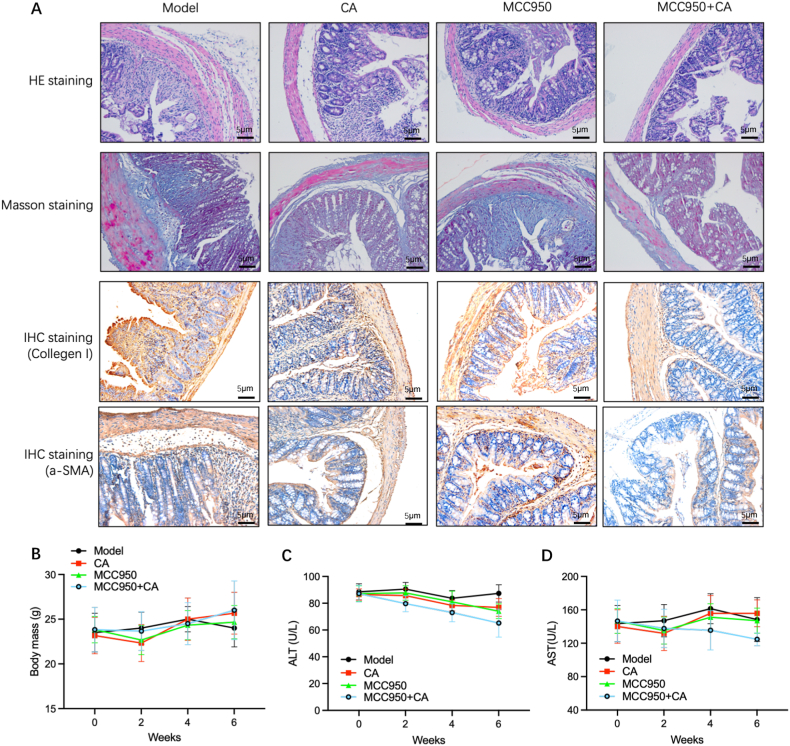
**Combination of Calycosin and NLRP3 inhibitor treating IBD in vivo**. (A) The pathohistological manifestations were analyzed by HE staining, fibrotic area was gaged by Masson staining and the expression of Collagen-1 or α-SMA by IHC staining was measured in model animals with MCC950, CA or their combination. (B) The body mass of different groups was calculated. (C–D) The liver function (the serum levels of ALT and AST) in the indicated timepoints was determined in model animals with MCC950, CA or combination; n = 6.

These findings mentioned above indicated a potential therapeutic value of calycosin in inhibiting NLRP3-induced IL-33/ST2 signaling in intestinal interstitial fibrosis context. Hence, we used the NLRP3 inhibitor-MCC950 and combined it with calycosin in IBD model mice to observe the efficacy of such a combination therapy in treating intestinal interstitial fibrosis. The result showed that compared to MCC950 (i.p., 10 mg/kg, 3 times/week) or calycosin single use, the combination of MCC950 and calycosin significantly reduced the extent of intestinal interstitial fibrosis, which displaying the improvement of pathohistological manifestations as indicated by HE staining, the reduction of fibrotic area as reflected by Masson staining and the decrease of Collagen-1 or α-SMA expressions as shown by IHC staining (Fig. 5A). Furthermore, it was also observed that there was no significant difference between the IBD model group and the MCC950 single or calycosin single or combination treatment groups (Fig. 5B–D).

## 4 Discussion

In recent years, with the use of new technologies, the role of inflammatory immune-related targets has become increasingly prominent in the field of organ fibrosis research, and the continued strengthening of natural products will provide new ideas and strategies for the future development of anti-fibrotic drugs [31,32]. In this study, we demonstrated the molecular mechanism of calycosin-regulated NLRP3-IL-33/ST2 signaling in intestinal fibrosis in IBD from the perspective of abnormal inflammatory immune regulation, and explored the value of calycosin in combination with targeted inhibitors of NLRP3 signaling in the treatment of IBD. The study will certainly clarify the anti-inflammatory immune value of calycosin, expand the existing therapeutic options of calycosin, and hopefully provide new perspectives for developing more effective and individualized treatment strategies for IBD fibrosis and related concomitant diseases.

Fibrosis can occur in a wide range of organs and is mainly caused by external factors or stimulation by endogenous immune factors. Of note, increase of fibrous connective tissue or decrease of parenchymal cells in the organ tissue can destroy the structure and function of the organ [33]. There are various theories as to the pathogenesis of organ fibrosis, in which inflammatory immune regulation is the main one [34]. Accumulating studies have confirmed that NLRP3 inflammasome is closely associated with the development of fibrosis in many organs, including the lung, liver, kidney and heart [34,35]. Fibrosis caused by inflammasome activation may be related to a number of factors, including cell type, inflammatory factors types and genetic variation in the autocrine or paracrine response of signalling molecules downstream of the inflammatory factors, or a combination of these factors [[36], [37], [38]]. However, the specific molecular mechanism is not fully clarified. In the study, it was found that calycosin can attenuate intestinal interstitial fibrosis and improve the inflammation status in TNBS-induced IBD model mice and, importantly, the effect of calycosin on expression of these fibrogenic factors was NLRP3-dependent. Therefore, an further in-depth study of the interactions between NLRP3 inflammasomes and other signalling pathways could provide a scientific basis for the pathogenesis of tissue fibrosis-related diseases and provide a reference for the development of new drugs.

Recently, the NLRP3-modulated IL-33 has been implicated in the pathogenesis of several diseases including interstitial fibrosis [39,40]. As an early mediator of inflammation, IL-33 can enhance inflammatory responses through binding to its receptor ST2 [41]. A variety of studies have shown that the IL-33/ST2 signaling plays an important role in the process of wound healing and tissue fibrosis [40,42]. Fibrosis usually occurs in the late stages of repair of chronic and/or recurrent tissue injury. In these tissues, there is a massive infiltration of inflammatory cells and continued activation of fibroblasts, but the repair capacity of mesenchymal stem cells is diminished, giving rise to fibrotic disease [42]. Despite the significant impact of fibrotic disease on human health, there are no recent effective treatments that directly address its pathogenesis. Recently, it has been demonstrated that IL-33 play key roles in type 2 cytokine-driven inflammation and fibrosis [43]; moreover, promote ischemia-reperfusion induced renal or hepatic fibrosis in mice [44,45]. However, questions about IL-33 are not yet particularly clear, such as why the IL-33/ST2 signaling is aberrantly expressed in fibrotic disease, what pathways affect other inflammatory factors, and what are the specific mechanisms of this signaling pathway in fibrotic disease? These are all questions that need to be furtherly investigated. In this study, we demonstrated that the calycosin-induced downregulation of NLRP3 can impede the activation of IL-33/ST2 signaling in intestinal epithelial cells and finally reduce inflammation and the secretion of pro-fibrotic factors in intestinal interstitial cells. Our finding suggested that NLRP3-induced IL-33/ST2 signaling pathway may play an important role in the crosstalk between intestinal epithelial cells and intestinal interstitial cells.

However, there are some limitations in the current study. Considering the multiple and complex effects of calycosin (such as anti-inflammation, anti-apoptosis and antioxidation), it is necessary to investigate the molecular mechanism underlying the role of calycosin in orchestrating the NLRP3-IL-33/ST2 signaling, which finally contributes to the development of IBD. Further research will provide a theoretical basis for the selection of new drugs for the treatment of fibrotic diseases and also provide new directions for the development of clinical anti-fibrotic drugs. Fortunately, our in vivo experiments further demonstrated that calycosin and NLRP3-IL-33/ST2 signaling inhibition presented synergistic effects on intestinal interstitial fibrosis.

## 5 Conclusion

**Fig. 6:**
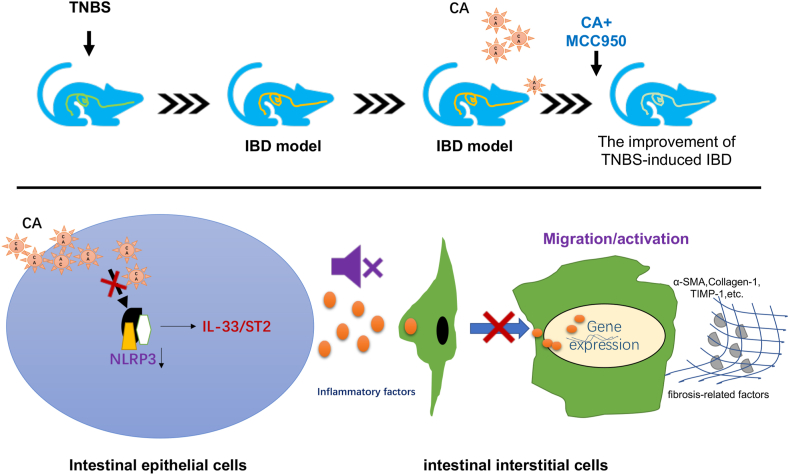
Schematic overview of the CA (Calycosin) mechanism for improving the intestinal interstitial fibrosis. Calycosin alleviated the symptom of TNBS-induced IBD model mice and improved intestinal interstitial fibrosis by downregulating NLRP3-IL-33/ST2 signaling and reducing inflammation and the secretion of pro-fibrotic factors.

Our present study demonstrated the value of calycosin in improving intestinal interstitial fibrosis by downregulating NLRP3-IL-33/ST2 signaling and finally reducing inflammation and the secretion of pro-fibrotic factors (Fig. 6). These findings extend previous studies and might provide the research evidence for therapeutic options of intestinal interstitial fibrosis. Of course, it is necessary to determine the feasibility and therapeutic benefit of calycosin on intestinal interstitial fibrosis based on a trial with large size samples in the future.

### Ethics approval

All animal studies (No. EACP/2021(06)/21) were approved by the Institutional Animal Care and Use Committees of The Second Affiliated Hospital, Zhejiang University School of Medicine, Hangzhou, Zhejiang.

## Funding

This project was supported by grants from the 10.13039/501100004731Natural Science Foundation of Zhejiang Province (No. LY21H270016). This research was supported by the Qingfeng Scientific Research Fund of the China Crohn's & Colitis Foundation (Grant No. CCCF-QF-2022B51-12).

## Data sharing statement

The data will not be shared as the data are considered property of The Second Affiliated Hospital, Zhejiang University School of Medicine, which does not allow sharing of data with third party. Nevertheless, the data will be available from the corresponding author on reasonable request.

## CRediT authorship contribution statement

**Xiujun Liao:** Writing – review & editing, Writing – original draft, Resources, Methodology, Funding acquisition, Data curation, Conceptualization. **Haiting Xie:** Validation, Resources, Methodology, Investigation, Formal analysis, Data curation. **Saojun Yu:** Writing – original draft, Methodology, Data curation, Conceptualization.

## Declaration of competing interest

The authors declare that they have no known competing financial interests or personal relationships that could have appeared to influence the work reported in this paper.

## Abbreviations

IBD: Inflammatory bowel disease
ECM: extracellular matrix
RA: Radix Astragali
CA: Calycosin
NLRP3: NOD-like receptor thermal protein domain associated protein 3
ASC: apoptosis-associated speck-like protein containing a CARD
TLR4: Toll-like receptor 4
IL-33: interleukin 33
ST2: soluble suppression of tumorigenicity 2
TNBS: 2,4,6-trinitrobenzenesulfonic acid
EDTA: Ethylene Diamine Tetraacetic Acid
BMDC: Bone marrow-derived dendritic cells
CK-8: Cell Counting Kit-8
DMEM: Dulbecco's modified Eagle medium
FBS: fetal bovine serum
FFPE: formalin-fixed paraffin-embedded
H2O2: hydrogen peroxide
HE: hematoxylin and eosin
PVDF: polyvinylidene fluoride
PCR: Polymerase chain reaction
ELISA: enzyme linked immunosorbent assay
TIMP: tissue inhibitor of metalloproteinase
α-SMA: alpha smooth muscle actin
KGF: keratinocyte growth factor
CTG: connective tissue growth factor
FGF2: fibroblast growth factor 2
IFG-1: insulin-like growth factor 1
PDGF-b: platelet derived growth factor subunit B
TGF-β: transforming growth factor-β
ALT: alanine aminotransferase
AST: aspartate aminotransferase
