# Supplementary material for: Calycosin prevents NLRP3-induced gut fibrosis by regulating IL-33/ST2 axis
Source: Heliyon. 2024 Apr 25;10(9):e30240. doi: 10.1016/j.heliyon.2024.e30240 (PMC11078877; doi:10.1016/j.heliyon.2024.e30240)
Supplement: Multimedia component 1 [file mmc1.docx]

84 inflammation-related cytokines and receptors in a mouse Inflammatory Cytokines & Receptors PCR Array

|  |  |
| --- | --- |
| Chemokines | C5, CCL1 (I-309), CCL11 (Eotaxin), CCL13 (MCP-4), CCL15 (MIP-1D), CCL16 (HCC-4), CCL17 (TARC), CCL2 (MCP-1), CCL20 (MIP-3A), CCL22, CCL23 (MPIF-1), CCL24 (Eotaxin-2), CCL26, CCL3 (MIP-1A), CCL4 (MIP-1B), CCL5 (RANTES), CCL7 (MCP-3), CCL8 (MCP-2), CX3CL1, CXCL1 (IL8RA), CXCL10 (INP10), CXCL11 (I-TAC/IP-9), CXCL12 (SDF1), CXCL13, CXCL2 (IL8RB), CXCL3, CXCL5 (ENA-78/LIX), CXCL6 (GCP-2), CXCL9. |
| Chemokine Receptors | CCL13 (MCP-4), CCR1, CCR2, CCR3, CCR4, CCR5, CCR6, CCR8, CX3CR1, CXCR1, CXCR2. Interleukins: IL13, IL15, IL16, IL17A, IL17C, IL17F, IL1A, IL1B, IL1RN, IL21, IL27, IL3, IL33, IL5, IL7, IL8, IL9. |
| Interleukin Receptors | IL10RA, IL10RB, IL1R1, IL5RA (CD125), IL9R. |
| Other Cytokines | AIMP1 (SCYE1), BMP2, CD40LG (TNFSF5), CSF1 (MCSF), CSF2 (GM-CSF), CSF3 (GCSF), FASLG (TNFSF6), IFNA2, IFNG, LTA (TNFB), LTB, MIF, NAMPT, OSM, SPP1 (Osteopontin), TNF, TNFSF10 (TRAIL), TNFSF11, TNFSF13, TNFSF13B, TNFSF4 (OX40L), VEGFA. |
| Other Cytokine Receptor | TNFRSF11B |
